# The Same Day on Repeat: A Unique Case of Persistent Déjà Vu Phenomenon as a Long COVID Symptom in an Older Adult

**DOI:** 10.1192/j.eurpsy.2023.1675

**Published:** 2023-07-19

**Authors:** I. Jileaeva, S. Bakre, L. Huff, B. Ratnakaran

**Affiliations:** 1 Virginia Tech Carilion School of Medicine; 2 Department of Psychiatry, Virginia Tech Carilion, Roanoke, United States

## Abstract

**Introduction:**

Déjà vu is a condition characterized by the experience of recognizing a current situation as familiar with the awareness that the recognition is inappropriate (Pasic *et al*. Psychiatria Danubina; 30, 21–25). This phenomenon has been well described in temporal lobe epilepsy, and is thought to be caused by abnormal synchronization in the corticolimbic network. While the etiology of neuropsychiatric symptoms in long COVID is still not well demarcated, studies have found that the virus attacks the temporal lobe and limbic system; therefore, we suggest that the ongoing symptoms of déjà vu, in this case, may be a manifestation of long COVID.

**Objectives:**

To illustrate a unique case of persistent déjà vu after severe infection with COVID-19 virus.

**Methods:**

The patient is a 79-year-old female with a pertinent past medical history of generalized anxiety disorder, major depressive disorder, and prior hospitalization for delirium who presented with a chief complaint of being tired of living the same day. The patient was hospitalized eight months prior for a severe COVID-19 infection that now requires continuous oxygen therapy. Since leaving the hospital, the patient began to develop episodic confusion, memory impairment, and tinnitus which gradually improved. However, the patient developed severe distress due to a constant feeling of déjà vu characterized by a sense of familiarity with events in her daily life. There was no history of loss of consciousness, abnormal involuntary movements, or other semiology related to seizures. Due to distress caused by the déjà vu symptoms, the patient also endorsed depression, anxiety, and insomnia, with deterioration of quality of life.

**Results:**

MRI showed mild volume loss and multifocal regions, including bilateral temporal lobes, of subcortical and periventricular high T2/FLAIR signal abnormality consistent with chronic white matter microangiopathy. MOCA score was 24/30, and the dissociative experiences scale score was 15.36/100. The patient continues to be treated for her depression, insomnia and anxiety with escitalopram 10mg daily and mirtazapine 7.5mg at night as a neuropsychological assessment and electroencephalogram are done.

**Conclusions:**

The incidence of neurological symptoms is more than 80% in severe cases of COVID-19 (Douaud *et al.* Nature; 604,697–707). This patient was likely predisposed to developing these symptoms due to her age and psychiatric history. Déjà vu occurs in temporal lobe epilepsy, schizophrenia, and depersonalization disorders, all of which affect the limbic-temporal lobe networks (Pasic *et al*. Psychiatria Danubina; 30, 21–25). Signal abnormalities in the medial temporal lobe are one of the findings seen on MRI in patients with neuropsychiatric symptoms after severe COVID-19 infection. We propose that the persistent déjà vu phenomenon along with cognitive impairments described in this case are manifestations of long COVID.

**Disclosure of Interest:**

None Declared

